# Supplementary material for: Heat-Killed Bifidobacterium breve B-3 Enhances Muscle Functions: Possible Involvement of Increases in Muscle Mass and Mitochondrial Biogenesis
Source: Nutrients. 2020 Jan 15;12(1):219. doi: 10.3390/nu12010219 (PMC7019314; doi:10.3390/nu12010219)
Supplement: Supplementary file 1 [file nutrients-12-00219-s001.pdf]

Supplemental Table 1. List of primer sets.

Target gene	Sequence (5' to 3') of forward primer	Sequence (5' to 3') of reverse primer	Reference
GAPDH	ACATTGTTGCCATCAACGAC	CTTGCCGTGGGTAGCGTCAT	Wang R., et al., Cell Metab. (2013) 17, 5, 685-694.
PGC-1α	AAAGGGCCAAGCAGAGAGA	GTAAATCACA CGGCGCTCTT	
COX1	TGGAGCCCCTGATATAGCATTC	CCAGCTTCTACTATGGAGGATGC	Kogot-Levin A., et al., PLoS One. (2016) 11, 10, e0165417.
COX2	GCACAATAGACGCCCAAGAAG	AATTCGTAGGGAGGGAAGGG	
COX4	TGAACAAGGGCACCAATGA	GCCATACACGTAGCTCTTCTC	
COX5B	TCCATACAATATGCTACCTCCAAA	ACAGATGCAGCCCACTATTC	

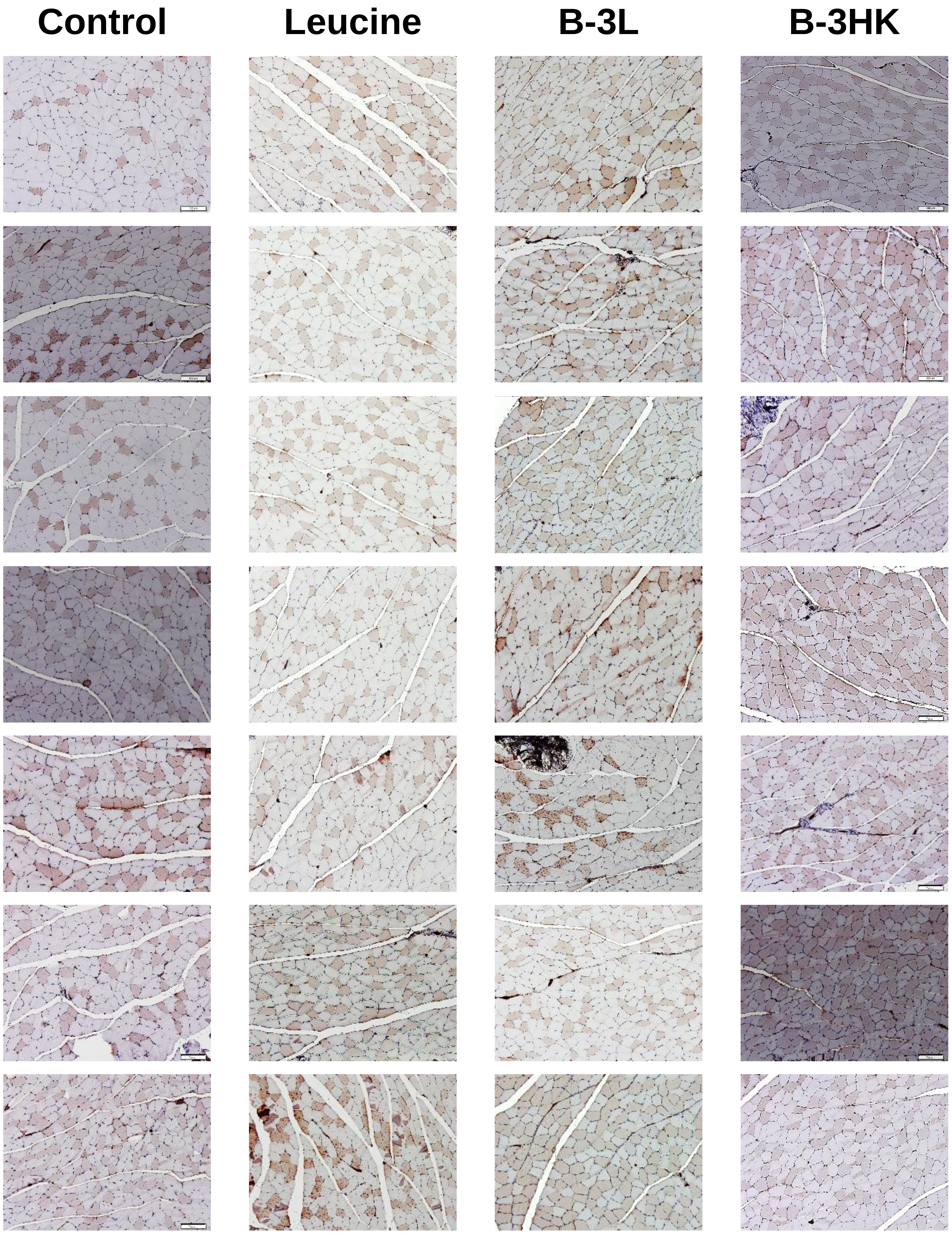

Supplemental Figure 1. Histopathological images

Representative image of immunohistochemistry against SERCA2 ATPase in the rat medial gastrocnemius (n=7).

Supplemental Table 2. Effects of B-3 on body and tissue weight in the treated mice at 4 weeks.

		Control	B-3HK	
Body weight (g)	initial	24.02 ± 0.23	24.04 ± 0.18	
	1 week	23.96 ± 0.21	24.21 ± 0.22	
	2 weeks	24.61 ± 0.28	24.48 ± 0.23	
	3 weeks	24.69 ± 0.25	24.49 ± 0.25	
	4 weeks	24.95 ± 0.28	24.56 ± 0.22	
Food intake (g)	initial	4.12 ± 0.45	5.23 ± 0.23	*
	1 week	4.82 ± 0.16	5.09 ± 0.17	
	2 weeks	3.80 ± 0.11	3.80 ± 0.13	
	3 weeks	4.06 ± 0.18	4.34 ± 0.17	
	4 weeks	3.60 ± 0.14	3.44 ± 0.13	
Liver weight (g)		1.20 ± 0.02	1.16 ± 0.03	
Liver weight / body weight (mg/g)		47.93 ± 0.69	47.68 ± 1.20	
Soleus weight (mg)		10.21 ± 0.49	11.98 ± 1.19	
Soleus weight / body weight (mg/g)		0.41 ± 0.02	0.49 ± 0.05	**

The data are presented as the mean and S.E.

Asterisks (*) denote significant differences from the control group at *:p<0.05 or **:p<0.01 (*t*-test).

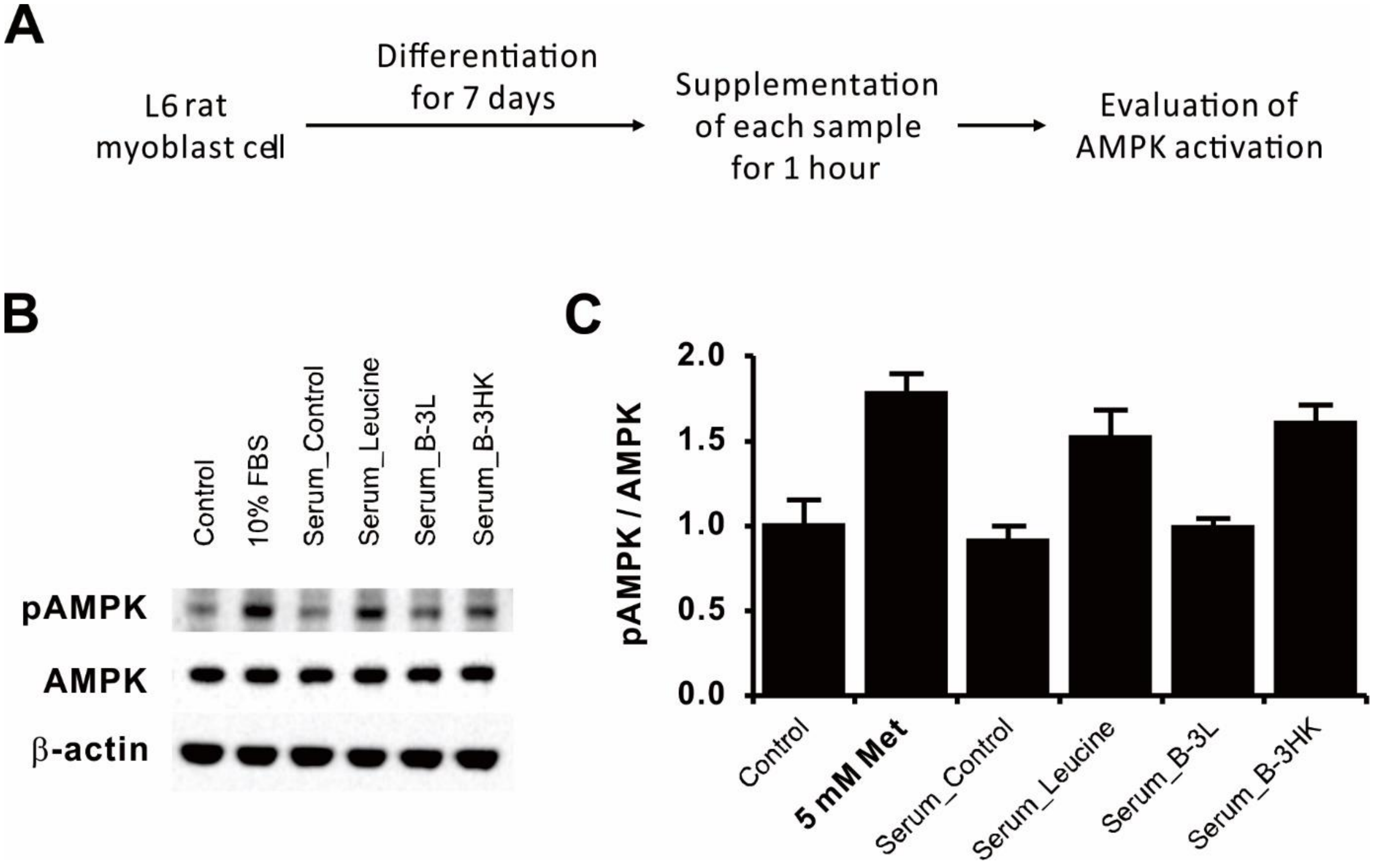

Supplemental Figure 2. Effects of the rat serum on AMPK activation *in vitro*

(A) Differentiated L6 cells were cultured with samples such as 5 mM metformin (Met, positive control) and 1% (v/v) rat serum for 1h, and then AMPK activation was determined using western blotting. (B) Representative bands and (C) the expression ratio of pAMPK to AMPK are indicated (n=3). The data are expressed as the mean and S.E.
